# Mitochondrial nicotinamide adenine dinucleotide hydride dehydrogenase (NADH) subunit 4 (*MTND4*) polymorphisms and their association with male infertility

**DOI:** 10.1007/s10815-021-02199-w

**Published:** 2021-04-24

**Authors:** Fatina W. Dahadhah, Mayyas Saleh Jaweesh, Mazhar Salim Al Zoubi, Manal Issam Abu Alarjah, Mohamad Eid Hammadeh, Houda Amor

**Affiliations:** 1 Department of Obstetrics & Gynaecology, Saarland University, Homburg, Saar Germany; 2 Department of Basic Medical Sciences, Faculty of Medicine, Yarmouk University, Irbid, 21163 Jordan

**Keywords:** mtDNA, MTND4, SNP, Male infertility

## Abstract

**Purpose:**

The purpose of the present study was to determine the relationship between infertility and the polymorphisms of mitochondrial NADH dehydrogenase subunit 4 (*MTND4*) by spermatozoa analysis in fertile and subfertile men.

**Methods:**

Samples were divided into 68 subfertile men (case group) and 44 fertile men (control group). After semen analysis, samples were purified. The whole genome was extracted using a QIAamp DNA Mini Kit and the mitochondrial DNA was amplified by using the REPLI-g Mitochondrial DNA Kit. Polymerase chain reaction (PCR) was used to amplify the MT-ND4 gene. Then, samples were purified and sequenced using the Sanger method.

**Results:**

Twenty-five single-nucleotide polymorphisms (SNPs) were identified in the *MTND4* gene. The genotype frequencies of the study population showed a statistically significant association between rs2853495 G>A (Gly320Gly) and male infertility (*P* = 0.0351). Similarly, the allele frequency test showed that rs2853495 G>A (Gly320Gly) and rs869096886 A>G (Leu164Leu) were significantly associated with male infertility (adjusted OR = 2.616, 95% CI = 1.374–4.983, *P* = 0.002; adjusted OR = 2.237, 95% CI = 1.245–4.017, *P* = 0.007, respectively).

**Conclusion:**

In conclusion, our findings suggested that male infertility was correlated with rs2853495 and rs869096886 SNPs in *MTND4*.

**Supplementary Information:**

The online version contains supplementary material available at 10.1007/s10815-021-02199-w.

## Introduction

Infertility, the inability to conceive after 12 months of unprotected sexual intercourse, affects 2.5–15% of couples in the world [1]. Approximately 50% of infertility cases are caused by male factors [2]. Sperm motility is an essential process for sperm movement to the fertilization site and normal fertilization which demand energy produced by sperm mitochondria [3]. Mitochondrial genes play an important role in the mature sperm construction and flagella movement after ejaculation [4].

Moreover, mitochondria, the powerhouse of the cell [5], have their own genome that encodes 13 proteins [6]. The human mitochondrial genome is considerably compact and circular consisting of 16,569 bp. The rate of mutations in the mitochondrial DNA (mtDNA) is high due to the lack of histones and DNA repair mechanisms [7]. Therefore, mutations that occur in the mitochondrial genome play a major role in some human genetic disorders [8]. It has been reported that mutations in the mtDNA are associated with certain types of male infertility. For instance, mutations in the mtDNA polymerase (POLG) locus were associated with male infertility [9]. In another study, a high incidence of single-nucleotide polymorphisms (SNPs) in mtDNA was observed in semen samples of poor sperm quality [10]. Moreover, several studies have shown that mtDNA mutations in sperm can lead to reduced sperm motility and eventually to male infertility [10**–**12]. However, mtDNA mutations especially reduce sperm motility without significantly compromising the fertility of young males [13].

Oxidative phosphorylation (OXPHOS) is an effective way to release energy; however, it also generates reactive oxygen species (ROS) during mitochondrial activity. Large amounts of ROS are detrimental to cells [14]. The generation of reactive oxygen species occurs as a natural physiological process in sperm. However, a small amount of ROS is actually required for normal sperm functioning. ROS such as nitric oxide (NO) and superoxide anion play an important role in the capacitation and the acrosome reaction. Moreover, ROS are involved in sperm-oocyte communication, but disproportionate levels of ROS production can negatively impact the spermatozoa quality and impair their fertilization capacity [15].

Several studies showed a strong correlation between impaired mtDNA and the occurrence of male infertility conditions such as asthenozoospermia, oligozoospermia, and teratozoospermia. For instance, large-scale deletions in the mtDNA have been identified in asthenozoospermia in various populations [16**–**18], while other studies established an association between CAG repeats and the development of oligozoospermia and teratozoospermia in infertile males [19].

Moreover, the removal of certain structural genes and tRNA genes may result in a large number of mtDNA deletions. Sperm containing defective mitochondria cannot effectively produce adenosine triphosphate (ATP) and are more likely to produce ROS and free radicals, thereby causing defects in mtDNA, producing less energy, and leading to deficits in motility and fertility [11, 20].

Genetic disorders are responsible for about 15–30% of male factor infertility. The proper understanding of the genetic basis of reproductive problems is requisite to managing the treatment of an infertile couple [21, 22]. Genetic disorders involving male infertility may be caused by chromosomal abnormalities, autosomal gene defects, or Y chromosome mutations.

The karyotype variant of Klinefelter’s syndrome, 47,XXY, is the most common numerical chromosomal abnormality associated with male infertility and occurs in 1 in 500 newborn males [23, 24]. Eleven percent of azoospermic and 0.7% of oligozoospermic males are probable to have this condition [24].

The human Y chromosome has also been found to be responsible for male infertility. Y-linked mutations have the greatest impact on spermatogenesis [23, 25]. Y-chromosome microdeletions have been reported in patients with suboptimal semen quality (7.4%) [26].

Sperms with defective mitochondria cannot effectively produce the required ATP and are more likely to produce free radicals/reactive oxygen species, causing genotoxicity and reduced sperm motility which can lead to male infertility [11, 20]. On the other hand, the mitochondrial respiratory chain consists of 13 proteins which are a constitutional part of membrane complexes. In particular, complex I includes seven nicotinamide adenine dinucleotide hydride (NADH) dehydrogenase subunits (*ND1*, *ND2*, *ND3*, *ND4*, *ND4L*, *ND5*, and *ND6*). In comparison, complex III contains cytochrome B, complex IV contains three subunits: cytochrome oxidase subunit I (*COX-I*), cytochrome oxidase subunit II (*COX-II*), and cytochrome oxidase subunit III (*COX-III*), and complex V contains *ATPase 6* and *ATPase 8* [27]. Complex I, the first enzyme of the electron transport chain, oxidizes NADH which is generated through the Krebs cycle in the matrix of mitochondria. Therefore, complex I is the main entry point for electrons to the respiratory chain and is suggested as the rate-limiting step in overall respiration [28].

*MTND4* gene spans from 10,760 to 12,137 in the human mtDNA genome (National Centre of Biotechnology Information; NCBI). *MTND4* is one of the core mitochondrial-encoded subunits of the mitochondrial membrane respiratory chain NADH dehydrogenase (Complex I) [29, 30]. *MTND4* gene codes for the NADH-ubiquinone oxidoreductase chain 4 (*ND4*) protein [31]. The *ND4* protein is located in the mitochondrial inner membrane and is the largest of the five complexes of the electron transport chain [32]. *MTND4* plays an important role in the oxidative phosphorylation process and it has been reported to be associated with sperm motility [10, 11, 33]. Various studies have shown a significant association of polymorphisms in *MTND4* with age-related muscular degeneration (AMD), Leber’s hereditary optic neuropathy (LHON), mesial temporal lobe epilepsy (MTLE), and cystic fibrosis [31], [34**–**36]. However, the overall association between *MTND4* polymorphisms and male infertility remains unknown.

Accordingly, we aimed to examine whether *MTND4* polymorphisms contribute to male infertility. We first identified SNPs in the *MTND4* gene by Sanger sequencing and then determined their association with infertility using a case-control study design.

## Materials and methods

### Sperm sample collection

One hundred and twelve semen samples were collected from subfertile and fertile males attending the in vitro fertilization clinic. The informed consent of all individuals was obtained before collecting samples. The study population aged between 26 and 48 years had been divided into two groups according to semen analysis results [37] to 68 subfertile and 44 fertile males. The individuals with age over 50 years old, men exposed to chemotherapy or radiotherapy, varicocele or any surgical procedure in the reproductive tract, diabetes, blood pressure and any chronic disease, hormonal imbalance, or Y chromosome microdeletion were excluded from this study.

### Mitochondrial DNA extraction

Before DNA extraction, semen samples were purified by discontinuous pure sperm gradient (45% and 90%) technique (Nidacon International, Sweden). Briefly, semen samples were loaded at the upper level of the gradient and centrifuged at 250*g* for 20 min. Thereafter, the pellet was collected and washed twice with a sperm washing medium. The absence of all other cells was confirmed by microscopic examination.

The whole genome was extracted from the spermatozoa using a QIAamp DNA Mini Kit; then, the mitochondrial DNA was amplified by using the REPLI-g Mitochondrial DNA Kit (QIAGEN, Hilden, Germany), as recommended by the kit instruction manual. Isolated DNA with an optimal density ratio of 260/280 of 1.8 or more was chosen for further analysis and stored at − 80 °C.

### Polymerase chain reaction

The polymerase chain reaction was applied to determine the gene variant by using self-designed pairs of specific primers using PRIME 3 software for the target gene (*MTND4*) (*Nd4*.F 5′-CTACGTACATAACCTAAACC-3′, *Nd4*.R 5′-CTGATGTTTTGGTTAAAC-3′ (Tm = 49 °C)), where the amplicon size is 1432 bp. An additional internal primer *Nd4*.I: 5′-CTTAAAACTAGGCGGCTATGG-3′ was designed for sequencing. The primers were based on the human mitochondrial sequence; accession number NC_012920, provided by the National Centre of Biotechnology Information and ordered from Microsynth seq lab-Germany.

**Fig. 1:**
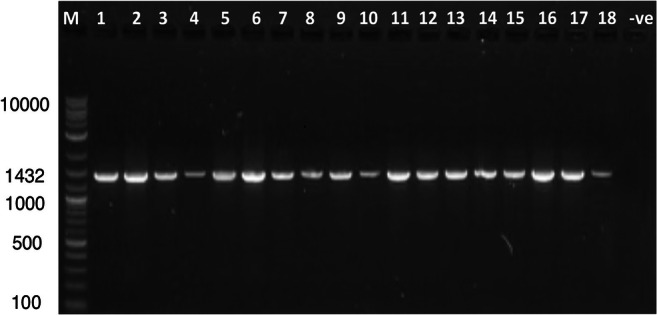
Representative gel electrophoresis on 1% agarose gel of PCR products for the amplification of the *MTND4* gene (1432 Bp). Lane M: DNA ladder (100–10,000 bp) (NE Biolabs, USA), lanes 1-18: PCR sample products, lane -ve: negative control. Electrophoresis was carried out at 100 V for 45 min. Gels were stained with red-safe stain and then DNA was visualized by ultra-violet (UV) transilluminator using Image LabTM Software (BIO-RAD, USA)

The amplification reaction was carried out in a 30 μL mixture using ThermoScientific Dream Taq Green PCR master mix (2×), according to manufacturer instructions. To confirm the presence of an amplified PCR product, 5 μL of each PCR sample was investigated by 1% agarose gel electrophoresis using 1× TBE buffer and a DNA ladder (1 kb) (NE Biolabs, USA) as a reference. Electrophoresis was carried out at 100 V for 45 min. Gels were stained with red-safe stain and then DNA was visualized by ultra-violet (UV) transilluminator with Image LabTM Software (BIO-RAD, USA) (Fig. 1).

### DNA sequencing

Samples were purified and sequenced by the Sanger method at Microsynth Seq lab in Germany. The SNPs of *MTND4* were detected by sequence analysis based on the reference sequence of human MT (GenBank accession number: NC_012920). The sequenced DNA samples were analyzed using Mutation Surveyor software to determine the mt-DNA variants.

### Statistical analyses

Genotypes and allele frequencies between the subfertile (case) and fertile (control) groups were performed using the chi-square test and Fischer’s exact test, respectively. The identified SNPs were also tested for the Hardy-Weinberg equilibrium test to determine the genotype frequencies and to describe statistically significant deviations from the equilibrium. The allele frequencies between the subfertile (case) and fertile (control) groups were measured according to odds ratios (ORs) and 95% confidence intervals (CIs). *P* value was considered statistically significant if *≤* 0.05. Statistical analyses were performed using the SPSS Version 22 for Mac.

## Results

In this study, males who had one child or more and had normal semen parameters [volume: 1.5 ml, sperm count: 15 million spermatozoa/ml; normal forms: 4%; vitality: 58% live; progressive motility: 32%; total (progressive + nonprogressive) motility: 40%, according to WHO guideline 2010] were considered as the fertile group, and those who failed to have children after 12 months or more of regular unprotected sexual intercourse and had one sperm parameters under WHO (2010) criteria were considered as the sub-fertile group. Thus, subjects were divided into two groups: a control group (fertile, *n* = 44) and a case group (subfertile, *n* = 68).

The study population did not show any significant difference between the ages of the subfertile and fertile group (*P* = 0.247). On the other hand, the semen analysis showed significant differences in the mean percentage of sperm concentration, total motility, and morphologically normal spermatozoa between the fertile and subfertile individuals (*P* < 0.0001) (Table 1).

**Table 1:** Comparison of the semen analysis parameters between the fertile and subfertile groups

Parameter	Fertile (*n* = 44) Median (range)	Subfertile (*n* = 68) Median (range)	*P* value
Age	34 (26–48)	34 (26–48)	0.247
Sperm concentration (106 × 1ml)	78.5 (17–185)	28 (0.6–135)	<0.0001
Total motility (PR + NP%)	67.5 (44–90)	48.5 (2–88)	< 0.0001
Morphologically normal spermatozoa (%)	24.5 (20–30)	15 (0–28)	< 0.0001

### Genotypes and allelic frequencies

A total of twenty-five SNPs in *MTND4* in the case and control groups were detected: rs2853495, rs2857284, rs2853496, rs2853497, rs3087901, rs2853493, rs2853490, rs3088053, rs2853491, rs2857285, rs28358282, rs28594904, rs28669780, rs28415973, rs28471078, rs55714831, rs28358283, rs75214962, rs28529320, rs2853494, rs28609979, rs28358286, rs28359168, rs28384199, and rs869096886 (Table 2).

**Table 2:** Genotype frequency of *MTND4* polymorphisms between subfertile and fertile groups

SNP	Contig position	Protein position	Genotype	Subfertile (*N*)	Fertile (*N*)	*P* value
rs2853495 G>A	11719	Gly320Gly	GG	25	8	**0.0351**
			GA	0	0	
			AA	43	36	
rs2857284 T>C	10873	Pro38Pro	TT	49	35	0.0995
			TC	2	4	
			CC	17	5	
rs2853496 G>A,C	11914	Thr385Thr	GG	56	40	0.597
			GA	3	1	
			AC	1	0	
			AA	8	3	
rs2853497 G>A	12007	.Trp416.Trp	GG	63	39	0.598
			GA	3	4	
			AA	2	1	
rs3087901 T>A,C,G	11944	Leu395Leu	TT	63	42	0.548
			TC	0	0	
			CC	5	2	
rs2853493 A>G	11467	Leu236Leu	AA	66	40	0.158
			AG	0	0	
			GG	2	4	
rs2853490 G>A	11176	Gln139Gln	GG	66	40	0.183
			GA	0	2	
			AA	2	2	
rs3088053 A>C,G	11812	Leu351Leu	AA	64	42	0.758
			AG	0	0	
			GG	4	2	
rs2853491 C>T	11335	Asn192Asn	CC	66	42	0.655
			CT	0	0	
			TT	2	2	
rs2857285 T>C,G	10915	Cys52Cys	TT	66	43	0.241
			TC	0	1	
			CC	2	0	
rs28358282 T>C	10810	Leu17Leu	TT	67	42	0.434
			TC	1	1	
			CC	0	1	
rs28594904 G>A,C	11016	Ser86Asn Ser86Thr	GG	67	42	0.434
			GA	0	1	
			AA	1	1	
rs28669780 C>A	11603	Leu282Met	CC	67	42	0.434
			CA	0	1	
			AA	1	1	
rs28415973 T>C	12091	Ile444Ile	TT	67	42	0.434
			TC	0	1	
			CC	1	1	
rs28471078 T>C	11722	Leu321Leu	TT	67	43	0.754
			TC	0	0	
			CC	1	1	
rs55714831 C>T	11332	Ala191Ala	CC	67	43	0.754
			CT	1	1	
			TT	0	0	
rs28358283 A>G	10819	Lys20Lys	AA	67	44	0.419
			AG	0	0	
			GG	1	0	
rs75214962 C>T	11197	Gly146Gly	CC	67	44	0.419
			CT	0	0	
			TT	1	0	
rs28529320 T>C	11485	Gly242Gly	TT	68	43	0.211
			TC	0	0	
			CC	0	1	
Rs2853494 A>G	11641	Met294Met	AA	68	43	0.211
			AG	0	0	
			GG	0	1	
rs28609979 T>C	11365	Ala202Ala	TT	68	44	-
			TC	0	0	
			CC	0	0	
rs28358286 C>T	11674	Thr305Thr	CC	68	43	0.211
			CT	0	0	
			CC	0	1	
rs28359168 A>G	11947	Thr396Thr	AA	68	43	0.211
			AG	0	0	
			GG	0	1	
rs28384199 C>A,G	11777	Arg340Ser Arg340Gly	CC	67	44	0.419
			CG	0	0	
			GG	1	0	
rs869096886 A>G	11251	Leu164Leu	AA	52	26	0.147
			AG	1	1	
			GG	15	17	

*SNP* single-nucleotide polymorphism

The significant results are in bold

To determine whether the variations of *MTND4* were related to infertility, we compared each of the genotypes and allele frequencies between the case and control groups. For the rs2853495, the mutant allele was higher than the wild-type allele frequency (*P* = 0.002) (Table 3) and the number of homozygous mutant types (AA) was higher than the homozygous wild type (GG) (*P* = 0.0351). Furthermore, the wild-type allele frequency in rs869096886 was significantly higher than the mutant allele frequency (*P* = 0.007). The remaining SNPs showed no significant difference in allele and genotype distribution among fertile and subfertile groups. Moreover, all SNPs were tested for the Hardy-Weinberg genotype frequency test. All of these SNPs showed a significant deviation from HWE (*P* < 0.0001).

**Table 3:** Allele frequency of *MTND4* polymorphisms between subfertile and fertile groups

SNP	Contig position	Protein position	Allele	Subfertile (*N*)	Fertile (*N*)	OR (95% CI)*	*P* value
rs2853495 G>A	11719	Gly320Gly	G	50	16	2.616 (1.374–4.983)	**0.002**
			A	86	72		
rs2857284 T>C	10873	Pro38Pro	T	100	74	0.5255 (0.264–1.044)	0.071
			C	36	14		
rs2853496 G>A,C	11914	Thr385Thr	G	115	81	0.496 (0.200–1.230)	0.145
			A	20	7		
rs2853497 G>A	12007	Trp416Trp	G	129	82	1.348 (0.437–4.155)	0.771
			A	7	6		
rs3087901 T>A,C,G	11944	Leu395Leu	T	126	84	0.6000 (0.18–1.97)	0.573
			C	10	4		
rs2853493 A>G	11467	Leu236Leu	A	132	80	3.300 (0.962–11.31)	0.066
			G	4	8		
rs2853490 G>A	11176	Gln139Gln	G	132	82	2.415 (0.661–8.817)	0.196
			A	4	6		
rs3088053 A>C,G	11812	Leu351Leu	A	128	84	0.761 (0.222–2.611)	0.768
			G	8	4		
rs2853491 C>T	11335	Asn192Asn	C	132	84	1.571 (0.382–6.456)	0.714
			T	4	4		
rs2857285 T>C,G	10915	Cys52Cys	T	132	87	0.379 (0.041–3.453)	0.650
			C	4	1		
rs28358282 T>C	10810	Leu17Leu	T	135	85	4.765 (0.487–46.58)	0.302
			C	1	3		
rs28594904 G>A,C	11016	Ser86Asn Ser86Thr	G	134	85	2.365 (0.386–14.45)	0.383
			A	2	3		
rs28669780 C>A	11603	Leu282Met	C	134	85	2.365 (0.386–14.45)	0.383
			A	2	3		
rs28415973 T>C	12091	Ile444Ile	T	134	85	2.365 (0.386–14.45)	0.383
			C	2	3		
rs28471078 T>C	11722	Leu321Leu	T	134	86	1.558 (0.215–11.27)	0.646
			C	2	2		
rs55714831 C>T	11332	Ala191Ala	C	135	87	1.552 (0.095–25.15)	1.000
			T	1	1		
rs28358283 A>G	10819	Lys20Lys	A	134	88	0.304 (0.014–6.411)	0.520
			G	2	0		
rs75214962 C>T	11197	Gly146Gly	C	134	88	0.304 (0.014–6.411)	0.520
			T	2	0		
rs28529320 T>C	11485	Gly242Gly	T	136	86	7.890 (0.37–166.44)	0.153
			C	0	2		
rs2853494 A>G	11641	Met294Met	A	136	86	7.890 (0.37–166.44)	0.153
			G	0	2		
rs28609979 T>C	11365	Ala202Ala	T	136	88	-	-
			C	0	0		
rs28358286 C>T	11674	Thr305Thr	C	136	86	7.890 (0.37–166.44)	0.153
			T	0	2		
rs28359168 A>G	11947	Thr396Thr	A	136	86	7.890 (0.37–166.44)	0.153
			G	0	2		
rs28384199 C>A,G	11777	Arg340Ser Arg340Gly	C	134	88	0.304 (0.01–6.41)	0.520
			G	2	0		
rs869096886 A>G	11251	Leu164Leu	A	105	53	2.237 (1.24–4.01)	**0.007**
			G	31	35		

*SNP* single-nucleotide polymorphism, *OR* odds ratio, *CI* confidence interval

The significant results are in bold

## Discussion

Sperm mitochondrial DNA contains no introns and lacks the protection of histones or DNA binding proteins. Therefore, it replicates rapidly without DNA repair mechanisms [6]. Consequently, mitochondrial mutation rates are higher by about 10–100 times than nuclear DNA. Mutations that occur in the mitochondrial genome play a major role in some human genetic disorders [8].

The purpose of the current study was to investigate whether polymorphisms in the *MTND4* gene are correlated with male infertility. Several studies have reported that among the identified *MTND4* SNPs, rs2853495 is related to ulcerative colitis and pancreatic cancer [38, 39], and rs869096886 is related to schizophrenia [40], whereas rs2857285 is associated with a more invasive form of ovarian cancer [41]. Moreover, rs28384199 is related to late-onset encephalopathy and is considered a highly pathogenic mutation [42].

Additionally, many males with ulcerative colitis, a principal form of inflammatory bowel disease, are unable to control their smoking, drinking, and eating habits, which can contribute to sexual dysfunction and infertility [43]. Moreover, it has been repeatedly reported that the fertility of schizophrenia patients is lower than that of people with other psychiatric illnesses and the general population [44].

In the present study, we scanned the polymorphisms of subfertile and fertile males by direct sequencing of the *MTND4* gene, and identified twenty-five SNPs as follows: rs2853495, rs2857284, rs2853496, rs2853497, rs3087901, rs2853493, rs2853490, rs3088053, rs2853491, rs2857285, rs28358282, rs28594904, rs28669780, rs28415973, rs28471078, rs55714831, rs28358283, rs75214962, rs28529320, rs2853494, rs28609979, rs28358286, rs28359168, rs28384199, and rs869096886. The rs28594904 (Ser86Asn or Ser86Thr), rs28669780 (Leu282Met), and rs28384199 (Arg340Ser or Arg340Gly) SNPs are missense variants, whereas the rest of SNPs are synonymous coding variants. All SNPs were also tested for the Hardy-Weinberg genotype frequency test (HWE). All of the SNPs showed a significant deviation from HWE (*P* < 0.0001) which means that the genotype distribution was not following Hardy-Weinberg and biased to one group.

Overall, in the genotype frequency test, we found a significant association between the SNP rs2853495 and male infertility. In the allele frequency test, rs2853495 (G11719A) and rs869096886 (A11251G) were also associated with male infertility, indicating that the presence of the allele itself may be associated with male infertility regardless of its genotype.

Moreover, the OR of rs2853495 SNP was associated with a 2.61 times increased risk of subfertile males than fertile ones. Furthermore, the OR of rs869096886 (2.237) was also higher for subfertile males than for control. These results demonstrated that although the rs2853495 and the rs869096886 SNPs are synonymous variants and do not cause an amino acid change, they can be related to male infertility. Nevertheless, synonymous variants have been suggested to play a role in gene regulation and possibly the development of diseases [45]. For instance, synonymous mutations have been reported to regulate gene expression through the miRNA [46]. In another example, a synonymous mutation was found to affect mRNA stability [47]. Therefore, these synonymous variants in the mtDNA need to be investigated by functional studies to reveal their possible role in sperm function and male infertility. Additionally, mtDNA methylation has been related to sperm quality which can be associated with the synonymous mutations [48]. The rs2853495 SNP is a synonymous variant, as the codon substitution from [GGG] to [GGA] at position 11719 does not change the encoded amino acid (glycine). Additionally, the rs869096886 is also a synonymous variant that is changed from [CTA] to [CTG] at position 11251. Therefore, the amino acid remains leucine which means that this change does not affect the resulting product’s protein sequence (NCBI).

Additionally, the copy number variant (CNV) is proposed to play a role in the development of certain diseases such as autism [49]. However, CNV will be more powerful when it is combined with other SNPs. Variants of unknown significance (VOUS) are another area that can be further investigated to correlate certain diseases and disorders with the genetic makeup of certain individuals. Genetic markers require intensive functional studies to reveal the molecular role of specific variants in the development of disease.

Less is known about mitochondrial gene polymorphisms in male infertility. Therefore, we identified the genetic associations between mitochondrial polymorphisms and male infertility. Our study is the first to explore the association between *MTND4* SNPs and male infertility. Although the sample size was small, our findings suggest that the rs2853495 and rs869096886 SNPs in *MTND4* might be associated with male infertility. However, analysis of a larger sample is needed and will allow a better understanding and clarification of the role of these *MTND4* SNPs in male infertility.

## Conclusion

In conclusion, we identified an as yet unknown association between mitochondrial gene polymorphisms in *MTND4* and male infertility. In the genotype frequency test, we found a significant association between the SNP rs2853495 and male infertility. In the allele frequency test, rs2853495 and rs869096886 were also associated with male infertility. This indicates that mitochondrial genetics might help to give a better understanding of the correlation between the presence of these SNPs and the male’s infertility. Moreover, larger prospective studies are required to confirm these associations of mitochondrial gene polymorphisms and male infertility and to clarify the definite effect of the mitochondrial genetic variations in male infertility.

## Supplementary Information

ESM 1 (DOC 490 kb)
