# Supplementary material for: Mitochondrial nicotinamide adenine dinucleotide hydride dehydrogenase (NADH) subunit 4 (MTND4) polymorphisms and their association with male infertility
Source: J Assist Reprod Genet. 2021 Apr 24;38(8):2021–9. doi: 10.1007/s10815-021-02199-w (PMC8417158; doi:10.1007/s10815-021-02199-w)
Supplement: Supplementary file 1 — (DOC 490 kb) [file 10815_2021_2199_MOESM1_ESM.doc]

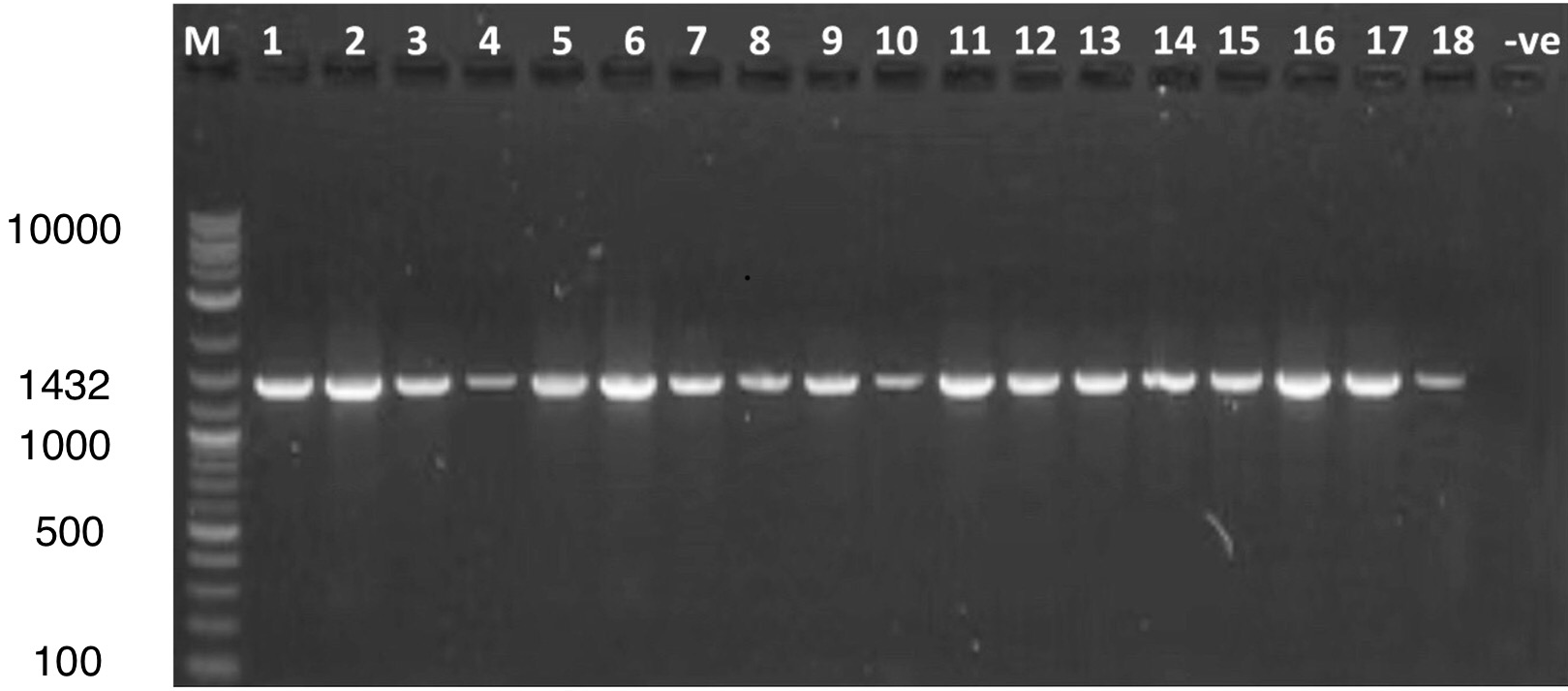


**Figure 1.** Representative gel electrophoresis on 1% agarose gel of PCR products for the amplification of the *MTND4* gene (1432 Bp). Lane M: DNA Ladder (100-10000 bp) (NE Biolabs, USA), Lane 1-18: PCR samples products, lane -ve: negative control. Electrophoresis was carried out at 100V for 45 min. Gels were stained with red-safe stain and then DNA was visualized by ultra-violet (UV) transilluminator using Image LabTM Software (BIO-RAD, USA).
